# Preoperative coagulation biomarkers associate with survival and pulmonary embolism after surgical treatment of non-spinal skeletal metastases

**DOI:** 10.1186/s12959-022-00431-w

**Published:** 2022-11-22

**Authors:** Sanna Matilainen, Gilber Kask, Jyrki Nieminen, Riitta Lassila, Minna Laitinen

**Affiliations:** 1 Department of Orthopedics and Traumatology, Lohja Hospital, Helsinki University Hospital, University of Helsinki, Helsinki, Finland; 2 Department of Orthopedics and Traumatology, Helsinki University Hospital, University of Helsinki, Helsinki, Finland; 3 Coxa, Hospital for Joint Replacement, Tampere, Finland; 4 Coagulation Disorders Unit, Department of Hematology, Comprehensive Cancer Center, Research Program Unit of Systems Oncology, Oncosys University of Helsinki, Helsinki, Finland; 5 Department of Orthopaedics and Traumatology, Bone Tumour Unit, HUS, Helsinki University Hospital, Topeliuksenkatu 5, P.O.Box 266, FI-00029, Helsinki, Finland

**Keywords:** Fibrinogen, FVIII, Pathological fracture, Thromboprophylaxis, Skeletal metastasis

## Abstract

**Background:**

Thrombotic complications are synergistic and associated with orthopedic procedures, trauma, and malignancy. Because cancer enhances coagulation activity and vice versa, we assessed preoperative biomarkers for survival and complications after treatment of pathologic fractures in non-spinal skeletal metastases.

**Patients/methods:**

Our study population comprised 113 actual or impending pathologic fractures in 100 patients admitted to two referral centers. Laboratory variables were collected retrospectively from patient records and analyzed related to incidence of pulmonary embolism (PE) and mortality (Kaplan-Meier and Cox regression analyses and biomarker quartiles).

**Results:**

Preoperative coagulation variables were high without exceptions. PE occurred in 12 patients at 36 post-operative days at incidence of 11% in the lower and 13% in the upper extremity fractures. Patients with fibrinogen exceeding 5 g/l (log-rank 0.022) developed PE earlier (5 to 15 days postoperatively) than others. Also, mean patient survival with normal fibrinogen range (2–4 g/l) was 34 months, whereas it halved upon elevated fibrinogen (log-rank *p* = 0.009). Survival in patients with FVIII levels under 326 IU/dl (Q3) was 22 months, but only 7 months if FVIII exceeded 326 IU/dl (log-rank *p* = 0.002). Combined elevated fibrinogen and FVIII predicted survival: for patients with levels below threshold limits was 22 months versus only 7 months when both variables exceeded the ranges (log-rank *p* < 0.001). Multivariate analysis to control confounders supported an independent role of fibrinogen and FVIII for survival.

**Conclusions:**

Our study has established fibrinogen and FVIII as potential preoperative contributors of survival and complications after treatment of metastatic fractures. These results highlight the need for novel anticoagulation and thromboprophylaxis strategies among these patients.

**Supplementary Information:**

The online version contains supplementary material available at 10.1186/s12959-022-00431-w.

## Introduction

Cancer, trauma, and orthopedic procedures are well known risk factors for thromboembolic complications [1–3]. In orthopedic surgery, the rate of venous thromboembolism (VTE) after major elective lower extremity surgery with postoperative thromboprophylaxis is estimated to be 1% [4–6]. Oncologic surgery further increases this risk, as the rate of VTE is 5% with postoperative thromboprophylaxis in abdominal cancer surgery [7]. In patients with surgically treated skeletal metastases, the incidence rate of thromboembolic complications is even higher, despite thromboprophylaxis. Their rate for deep venous thrombosis (DVT) is 2–11%; and that of pulmonary embolism (PE) 2–9%, and for lethal PE under 1% to over 3% [1, 8–11]. The overall postoperative mortality rate is high, ranging from more than 7% during the first 30 days to between 38 and 47% at 1 year [8–12].

Cancer leads to an imbalance in coagulation and hemostasis, which further contributes to cancer progression. The clinical manifestations of this imbalance range from DVT and PE to hemorrhagic complications [13]. Preoperative laboratory tests before major, hemostatically risky surgery usually include complete blood count, prothrombin time (PT) as a marker of vitamin K-dependent clotting factors, and activated partial thromboplastin time (APTT), which reflects the intrinsic pathway. Fibrinogen is the most abundant coagulation protein and the source of fibrin formation, and may prolong thrombin time (TT) in cancer-associated dysfibrinogenemia. Coagulation factor VIII (FVIII) and fibrinogen as acute phase reactants are associated with a risk for thrombosis and its reoccurrence, especially in cancer patients [2, 14, 15].. D-dimer depicts fibrin turnover, depending on both coagulation and fibrinolysis, and is often elevated in cancer patients, especially in association with chemotherapy-linked VTE [16].

FVIII, fibrinogen, and D-dimer have recently all been reported to improve diagnostics and aid surgical decision-making in pancreatic [17–19] and gynaecological cancers [20]. Therefore we aimed at identifying associations between coagulation biomarkers and postoperative thromboembolic complications and survival in patients who had undergone surgery for non-spinal actual or impending pathologic fractures. We targeted the potential role of biomarkers to aid in surgical decision-making, and to consider novel thromboprophylactic strategies.

## Patients and methods

Patients were identified from two referral bone tumor centers in Finland. All were operatively treated for non-spinal skeletal metastases due to actual or impending pathologic fractures between March 20th, 2016 and November 14th, 2020. An actual fracture has occurred, and an impending fracture has a degree of bone disruption which warrants prophylactic surgical stabilization to prevent a fracture, intractable pain, or the loss of ambulatory ability. Data from all consecutive patients were collected retrospectively from medical records. The study was approved by the institutional ethical review boards (HUS/234/2020, 14.1.2020 Helsinki, Finland).

All blood and plasma samples were collected preoperatively as part of routine diagnostic laboratory examinations, and analyzed at Helsinki University Hospital Laboratory HUSLAB, and Tampere University Hospital Laboratory FimLab. The following variables were captured: 3. A traditional subcutaneous low-molecular-weight heparin (LMWH) dose, either enoxaparin 40 mg or tinzaparin 4500 IU once daily was administered for 30 days. The laboratory variables included blood cell counts (of red blood cells, leukocytes and platelets), creatinine, and a coagulation panel with PT, TT, APTT, fibrinogen, FVIII, antithrombin, and D-dimer. The analysis covered both the whole group, and the most common primary diseases (multiple myeloma, breast, kidney and lung cancers).

### Coagulation assays

Citrated 3.2% plasma was analyzed for PT with Nycotest PT (Axis-Shield PoC As, Oslo, Norway) and APTT with Actin FSL (Siemens Healthcare Diagnostics, Marburg, Germany, also providing the following reagents). Fibrinogen levels were determined using Multifibren U, and BC Thrombin Reagent was used for TT. FVIII was analyzed with a one-stage clotting assay (Pathromtin SL and Coagulation Factor VIII Deficient Plasma), and antithrombin (AT) was measured with a chromogenic assay (Berichrom Antithrombin III). D-dimer levels were captured with an immunoturbidimetric assay (Tina-quant D-dimer; Roche Diagnostics, Mannheim, Germany).

### Statistical analysis

All statistical analyses were conducted using IBM SPSS Statistics version 27. Kruskal-Wallis analysis with post hoc Dunn test was used to identify differences in laboratory variables between groups. The biomarker levels were analyzed according to the reference levels and interrange quartiles, and dichotomized for further analysis for survival time. Multivariate logistic regression analysis was performed to control for confounding variables (modified Charlston comorbidity index (CCI), chemotherapy, radiotherapy, age at operation, hypertension, fracture) and to identify risk factors for postoperative survival. Survival and occurrence of PE were assessed with the Kaplan-Maier method with a log-rank test, and Cox regression analysis controlling for the confounding variables (as above) was used to identify variables affecting both survival and PE. A *P*-value < 0.05 was used to indicate statistical significance.

## Results

### Characteristics of patients with operatively treated pathologic fractures

A total of 113 pathologic actual (*n* = 94, 83%) or impending (*n* = 19, 17%) fractures were treated surgically in 100 patients (Table 1).

**Table 1:** Characteristics of patients with upper and lower limb metastases

	Fractures/ Patients	Mean age at operation in years (range)		Primary disease (number, %)	Site (number, %)
Upper limb	16/15	69 (32–96)	76 (57–89)	Kidney cancer (5, 33) Breast cancer (4, 27) Lung cancer (2, 13) Multiple myeloma (1, 7) Leiomyosarcoma (1, 7) Prostate cancer (1, 7) Unknown primary disease (1, 7)	Diaphyseal humerus (9, 56) Proximal humerus (6, 38) Distal humerus (1, 6)
Lower limb	97/92		68 (32–96)	Breast cancer (24, 26) Kidney cancer (21, 23) Lung cancer (12, 13) Multiple myeloma (10, 11) Colon cancer (4, 4) Prostate cancer (4, 4) Squamocellular cancer (4, 4) Leiomyosarcoma (2, 2) Uterine cancer (2, 2) Rectal cancer (2, 2) Lymphoma (1, 1) Pancreatic cancer (1, 1) Ventricular cancer (1, 1) Melanoma (1, 1) Urothelial cancer (1, 1) Clear cell sarcoma (1, 1)NET (1, 1)	Proximal femur (40, 41) Femoral neck (19, 20) Acetabulum (15, 16) Distal femur (11, 11) Diaphyseal femur (7, 7) Proximal tibia (3, 3) Multiple femoral lesions (1, 1) Diaphyseal tibia (1, 1)

*NET* neuroendocrine tumor

Mean age of the patients at the time of operation was 69 years (range, 32–96 years), 54 patients were female and 46 male, and the most common primary diseases were multiple myeloma, breast, kidney, and lung cancers (Table 1). Thirty-seven patients had only skeletal metastasis, and 36 patients had both skeletal and pulmonary metastases, 8 patients had skeletal and liver metastases, and 16 patients had skeletal, pulmonary, and liver metastases. Mean CCI was 8.4 (median 8.0, range 6–13). Eleven patients received radiotherapy and 28 chemotherapy.

Mean postoperative survival was 17 months (median 6 months), and the survival at 1 month was 87%, at 3 months 61%, at 6 months 45%, at 1 year 39%, and at 3 years 14% (Table 2). Any major bleeding complications, such as requiring revision surgery or other intervention did not occur, neither minor bleeding complications were documented.

**Table 2:** Fibrinogen, FVIII, and D-dimer by survival time

Survival time	Fibrinogen (g/l) Mean/median (range)	FVIII (IU/dl) Mean/median (range)	D-dimer (mg/l) Mean/median (range)
under 1 month (*n* = 12)	4.9/5.1 (2.3–6.6)	361/353 (206–544)	3.7/5.0 (0.2–5.7)
1–3 months (*n* = 26)	6.1/5.8 (3.4–10.1)	282/247 (144–457)	2.9/2.8 (0.3–5.0)
3–6 months (*n* = 16)	6.4/6.6 (2.9–9.8)	213/198 (136–334)	3.0–2.3 (1.4–6.9)
6–12 months (*n* = 6)	6.4/6.7 (3.8–8.9)	263/257 (171–366)	1.4/1.3 (0.5–2.7)
1–2 years (*n* = 14)	4.7/5.0 (0.5–7.2)	228/240 (61–343)	2.1/1.8 (0.3–5.5)
2–3 years (*n* = 11)	4.6/4.7 (2.7–6.0)	215/197 (128–307)	2.5/2.4 (0.3–5.0)
Over 3 years (*n* = 14)	5.3/5.5 (0.4–8.4)	216/217 (86–365)	2.6/2.1 (0.7–5.0)

### Blood cell counts

We examined the differences in the routine laboratory variables, including complete blood cell counts and coagulation screens, between the most common primary malignant diseases (multiple myeloma, breast, kidney and lung cancers) compared with the other cancer types (Table 3). In general, basic blood cell count did not vary between patients or primary disease groups, except leukocyte count in patients with kidney and lung cancer, red blood cell count in patients with breast cancer, and platelet count in patients with multiple myeloma. One patient with multiple myeloma and subtrochanteric femoral fracture had a low hemoglobin count at admittance (64 g/l), partly caused by the fracture. The hemoglobin count increased to 98 g/l by red cell transfusion prior to the operation. This same patient also presented with a prolonged thrombin time of over 140 s, signifying cancer-associated dysfibrinogemia.

**Table 3:** Laboratory variables in all patients and patients with PE and some cancer types

Biomarker (normal range)	All patients mean/median (range), number	PE patients mean/median (range), number	Multiple myeloma mean/median (range), number	Breast cancer mean/median (range), number	Kidney cancer mean/median (range), number	Lung cancer mean/median (range), number	***P***-value
PT (70–130%)	85/84 (16–133), *n* = 96	87.4/87.0 (47–115), *n* = 10	83/87 (39–106), *n* = 11	92/91 (62–133), *n* = 25	77/78 (16–131), *n* = 23	88.5/93.0 (32.0–121.0), *n* = 13	ns
APTT (28–37 s)	33/32 (24–46), *n* = 89	31.4/30.5 (25–46), *n* = 10	29.9/29.0 (25–35), *n* = 11	30.1/29.0 (24–39), *n* = 25	35/35 (27–46), *n* = 19	34/32 (28–46), *n* = 19	Myeloma: 0.046 Breast ca: 0.001 Kidney ca: 0.033
Thrombin time (17–25 s)	18/16 (14–140), *n* = 86	17/16 (15–21), *n* = 11	29.3/16.5 (15–140), *n* = 10	17/16 (14–23), *n* = 25	16/16 (14–19), *n* = 18	16/16 (15–18), *n* = 11	ns
Fibrinogen (2–4 g/l)	5.5/5.5 (0.5–10.1), *n* = 87	6.1/6.2 (4–8), *n* = 10	4.6–5.0 (0.7–6.8), *n* = 11	4.8/4.4 (2.3–8.0), *n* = 25	6.4/6.5 (3.4–9.8), *n* = 18	5.8/5.8 (4.0–7.1), *n* = 11	Myeloma: 0.043 Breast ca: 0.013 Kidney ca: 0.025
Antithrombin (85–125%)	99/98 (39–270), *n* = 85	120/111 (77–270), *n* = 10	98/100 (86–116), *n* = 9	100.6/102.5 (66–140)	100/99 (81–121), *n* = 18	96/92 (79–124), *n* = 11	ns
D-dimer (< 0.5 mg/l)	2.7/2.3 (0.2–6.9), *n* = 88	2.9/3.0 (1–5), *n* = 11	2.4–2.1 (0.2–5.0), *n* = 11	2.9/2.3 (0.5–5.7), *n* = 25	2.0/2.1 (0.3–5.0), *n* = 18	3.0/2.5 (1.4–5.0), *n* = 11	ns
FVIII (60–160 IU/dl)	250/223 (61–544), *n* = 62	295/270 (195–457), *n* = 7	258/287 (86–366), *n* = 7	240/226 (107–544), *n* = 16	239/213 (159–425), *n* = 13	243/214 (178–374), *n* = 9	ns
Leuc (3.4–8.2 E9/l)	8.1/7.6 (1.1–22.2), *n* = 100	9.6/9.6 (5.7–14.9), *n* = 2	6.9/6.3 (3.1–12.4), *n* = 11	7.3/7.6 (3.0–12.9), *n* = 27	8.2/8.0 (3.1–15.1), *n* = 23	11.2/10.1 (5.8–18.2), *n* = 13	Lung ca: 0.000
Hgb (134–167 g/l)	113/113 (64–151), *n* = 100	116/118 (89–150), *n* = 12	106/101 (64–150), *n* = 11	120/123 (98–142), *n* = 27	109/105 (78–144), *n* = 23	114/113 (87–139), *n* = 13	Breast ca: 0.014
PLT 150–360 E9/l)	267/267 (46–636), *n* = 100	275/292 (80–499), *n* = 12	201/159 (80–402), *n* = 11	238.9/243.0 (46–414), *n* = 27	301/286 (123–636), *n* = 23	280/290 (169–414), *n* = 13	Myeloma: 0.022
Crea (60–100 umol/l)	86/68 (32–776), *n* = 99	84/78 (46–181), *n* = 12	120/85 (58–361), n = 11	61/61 (32–112), n = 27	127/89 (50–776), *n* = 23	72/61 (41–159), *n* = 13	Myeloma: 0.033 Breast ca: 0.003 Kidney ca: 0.000

*Hgb* hemoglobin; *PLT* Platelet count; *ns* nonsignificant; *ca* cancer

### Fibrinogen

Mean fibrinogen was high in all patients (reference range 2–4 g/l, mean in all 5.5 g/l). Compared with the other cancer types, fibrinogen levels were the lowest in patients with multiple myeloma (*p* = 0.043) and breast cancer (*p* = 0.013), whereas the highest in patients with kidney cancer (*p* = 0.025) (Table 3).

### APTT, FVIII, and D-dimer

APTT (reference range 28–37 s, mean 33 s) was shorter in multiple myeloma (*p* = 0.046) and breast cancer (*p* = 0.001), but longer in kidney cancer (*p* = 0.033) than in other patients.

Overall, FVIII and D-dimer levels were quite similarly elevated (Table 3). In all patients, mean FVIII was 250 IU/dl (reference range 60–160 IU/dl), and mean D-dimer was 2.7 mg/l (reference range < 0.5 mg/l), without differences between primary disease groups.

### Incidence and timing of postoperative PE in patients with upper and lower extremity fractures

The overall incidence of PE was 12% (12/100 patients) (Table 4). PE occurred equally in patients with lower extremity fractures (11% (*n* = 10/92)) and upper extremity fractures (13% (*n* = 2/15)). Eleven patients had an actual pathologic fracture and one had an impending pathologic fracture. Of all patients with PE, six (50%) had only skeletal metastases, four (33%) had skeletal and pulmonary metastases, one (8%) had skeletal and liver metastases, and one (8%) skeletal, pulmonary, and liver metastases. Two patients with only skeletal metastasis had actual humeral fractures, one patient with multiple myeloma and the other with lung cancer.

**Table 4:** Characteristics of patients with postoperative PE

Primary disease (number, %)	Mean age in years at the time of operation (range)	Fracture site (number, %)	
Lung cancer (4, 33) Breast cancer (3, 25) Colon cancer (1, 8) NET (1, 8) Ventricular cancer (1, 8)n Multiple myeloma (1, 8) Uterine cancer (1, 8)	70 (58–79)	Upper limb (2, 13)	Proximal humerus (2, 17)
		Lower limb (10, 11)	Proximal femur (4, 33) Femoral neck (2, 17) Acetabulum (2, 17) Distal femur (2, 17)

*NET* neuroendocrine tumor

On average, postoperative PE in 12/100 patients occurred at the postoperative day 36 (range 0–141 days). The occurrence of PE was not associated with primary disease, number of tissues with metastases, or fracture type. An intraoperative saturation drop was recorded in four patients with PE. In one patient PE manifested on the first postoperative day. The patient had a history of DVT and preoperative anticoagulation, and two metastases were operated on the same day: a pathologic femoral neck fracture and a contralateral impending femoral diaphyseal fracture, while all others had one fracture operated at a single setting.

Records of postoperative thromboprophylaxis were available for 95 patients. While 92 patients received thromboprophylaxis, three patients with upper limb fractures were not administered with thromboprophylaxis. Of the three patients who did not get thromboprophylaxis, one manifested with postoperative PE.

Two patients died of overt PE. One patient with lung cancer and humeral pathologic fracture died at 63 days. The other patient with breast cancer and a distal femoral fracture died at postoperative day 9, showing already an intraoperative saturation drop. The first patient with upper limb fracture did not have either pre- or postoperative thromboprophylaxis, but the other patient had both forms of prophylaxis. All the other patients developed PE under postoperative thromboprophylaxis, and seven out of 12 patients also had preoperative thromboprophylaxis. In this dataset, however, thromboprophylaxis did not signify the onset of PE (Kaplan-Meier log-rank *p* = 0.339). Moreover, the occurrence of PE did not influence postoperative survival either in univariate or multivariate models (Kaplan-Meier, log-rank *p* = 0.998, Cox analysis (RR 0.825, CI 95% 0.442–1.538)).

### Fibrinogen and FVIII as biomarkers of postoperative survival and PE

**Fig. 1:**
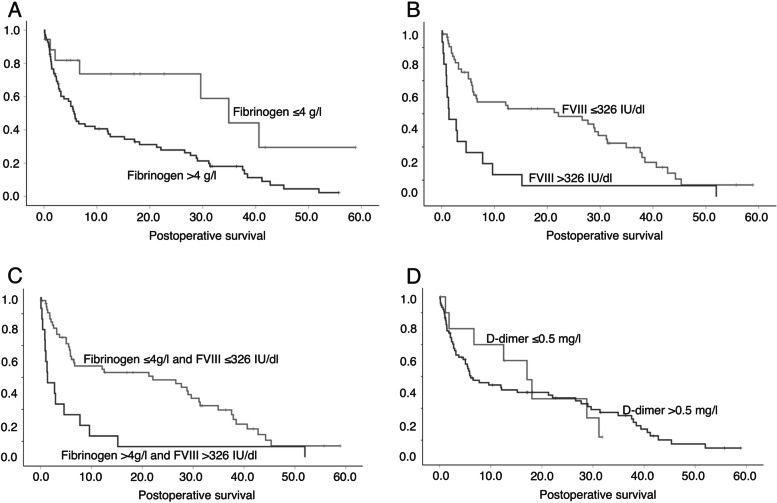
Kaplan-Meier survival analysis showing postoperative survival (months) relative to coagulation variables **A** Kaplan-Meier survival analysis of all patients, comparison of survival between patients with fibrinogen > 4 g/l and ≤ 4 g/l (log rank *p* = 0.009). **B** Kaplan-Meier survival analysis of all patients, comparison of survival between patients with FVIII > 326 IU/dl and ≤ 326 IU/dl (log-rank *p* = 0.002). **C** Kaplan-Meier survival analysis of all patients, comparison of survival between patients with both fibrinogen > 4 g/l and FVIII > 326 IU/dl, and patients with fibrinogen ≤4 g/l and/or FVIII ≤325 IU/dl (log-rank *p* = < 0.001). **D** Kaplan-Meier survival analysis of all patients, comparison of survival between patients with D-dimer > 0.5 mg/l and ≤ 0.5 mg/l (log-rank *p* = 0.905)

The results of preoperative fibrinogen and FVIII as biomarkers for postoperative survival were significantly predictive. Mean postoperative survival in patients with fibrinogen at normal range (*n* = 18, 2–4 g/l) was 34 months (median 35 months), but it was less than half (15 months) in patients whose fibrinogen levels were higher than the upper normal limit of 4 g/l (*n* = 69, median 6 months) (log-rank *p* = 0.009) (Fig. 1A).

In general, FVIII was high (mean 250 IU/dl), and the level of the highest interrange quartile 326 IU/dl was chosen as the cut-off level for analysis. Mean postoperative survival in patients with FVIII levels below 326 IU/dl (*n* = 47) was 22 months (median 21 months), while only 7 months (median 1 month) in those with FVIII exceeding 326 IU/dl (Q3) (*n* = 15, log-rank *p* = 0.002) (Fig. 1B).

The combination of fibrinogen exceeding 4 g/l and FVIII exceeding 326 IU/dl predicted postoperative survival. Thus, in patients with both or either variable below the threshold (*n* = 55), the mean survival rate was 22 months (median 22 months), whereas it was only 7 months in those whose both variables exceeded the reference ranges (*n* = 15, median 1 month) (log-rank *p* < 0.001) (Fig. 1C).

Overall, these laboratory variables depicted significant results in the Kaplan-Maier analysis, and were subjected to further analysis with the Cox regression model and multivariate logistic regression analysis. Again, the multivariate Cox regression model showed significant results for both fibrinogen and combination of fibrinogen and FVIII after controlling for potentially relevant confounding variables (CCI, chemotherapy, radiotherapy, age over 65 years, hypertension, fracture, Table 5, additional file 1). Similarly, in logistic regression analysis the combination of high fibrinogen and FVIII was independently associated with survival longitudinally over 1 month (OR 8.3, 95% CI 1.7–40.6, *p* = 0.009), 3 months (OR 6.2, 95% CI 1.8–21.3, *p* = 0.004), 6 months (OR 3.6, 95% CI 1.0–12.7, *p* = 0.048), 1 year (OR 6.8, 95% CI 1.4–32.9, *p* = 0.018), and over 2 years (OR 8.9, 95% CI 1.1–72.8, *p* = 0.041). However, high D-dimer did not predict survival either in multivariate or univariate analysis (Fig. 1D, Table 5).

**Table 5:** Cox regression survival analysis of fibrinogen and FVIII levels and their combination after controlling confounding variables

	RR	95 CI	*p*-value
**Fibrinogen > 4 g/l**	2.63	1.14–6.09	0.023
**FVIII > 326 IU/dl**	3.12	1.55–6.28	0.001
**Hypertension**	1.96	1.03–3.74	0.040
**Fibrinogen > 4 g/l and FVIII > 326 IU/dl**	3.33	1.68–6.62	0.001
**D-dimer > 0.5 mg/l**	0.63	0.26–1.55	0.318

**Fig. 2:**
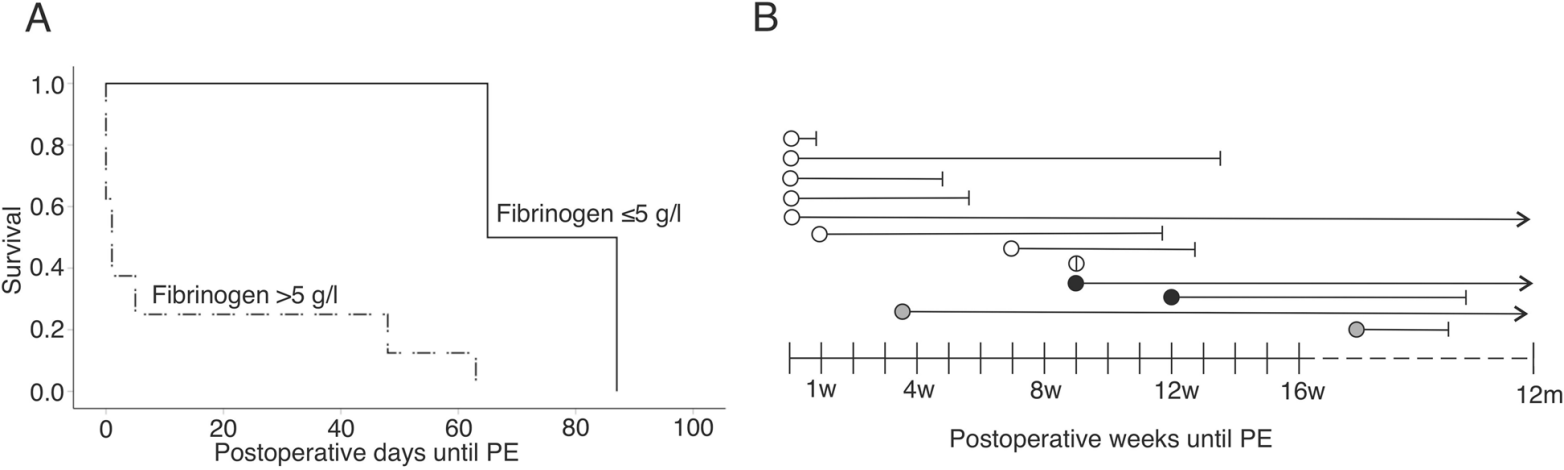
Timing of postoperative PE in relation to fibrinogen levels. **A** Kaplan-Meier survival analysis of patients with PE, patients with fibrinogen > 5 g/l manifested with PE earlier than patients with fibrinogen ≤5 g/l (log-rank *p* = 0.022). **B** Timeline of 12 patients with PE. Each line represents an individual patient and their postoperative survival, and circles indicate onset of PE. White circles indicate patients with fibrinogen > 5 g/l, black circles indicate patients with fibrinogen ≤5 g/l, gray circles patients with unknown fibrinogen level.

In contrast to survival, none of the preoperative biomarkers (fibrinogen, FVIII, or D-dimer did not predict) the overall occurrence of postoperative PE. Of the 12 patients who developed postoperative PE, fibrinogen was available in 10 patients, FVIII in 7 patients, and D-dimer in 11 patients. Fibrinogen predicted the earlier timing of PE at levels which exceeded 5 g/l (log rank *p* = 0.022) (Fig. 2A), while Cox regression analysis did not provide significance in risk (RR 0.019, 95% CI 0.000–10.7, *p* = 0.221). Those patients whose fibrinogen level was 5 g/l or less (*n* = 2) had PE later at 76 days (mean) (median 65 days) than the patients whose fibrinogen exceeded 5 g/l (*n* = 8) whose PE occurred at 15 days (median 1 days) (Fig. 2B). PE did not influence postoperative survival.

## Discussion

In this retrospective cohort study, 100 patients with 113 pathologic fractures treated operatively were studied to identify potential coagulation biomarkers for postoperative survival and complications. Preoperative fibrinogen and FVIII have been proposed as biomarkers and contributors for progressive primary malignant disease [18, 21, 22], and our findings further extend these observations to survival in patients having pathologic fractures. Since coagulation activity and fibrin generation may remain non-overt, i.e. without overt thrombosis, the preoperative coagulation biomarkers could reveal the underlying enhanced interactive mechanisms between coagulation and cancer. Therein, our results highlight the need for an overall improvement in anticoagulant strategies in patients who undergo surgery of pathologic fractures, including the upper extremity. Beyond this strong cancer-associated coagulation activity, PE associated only with fibrinogen levels only in its earlier appearance post surgery.

The role of the biomarkers of coagulation activity in patients with pathologic fractures has not previously been studied. In the study by Mattila et al. on biomarkers of coagulation in pancreatic tumors, the preoperative acute phase reactants fibrinogen and FVIII distinguished cancer from benign tumors [17, 18, 20]. Our data on pathological fractures showed that these coagulation biomarker levels are significantly increased in all patients. Indeed, fibrinogen levels exceeding normal ranges (4 g/l) were associated with decreased postoperative survival, and fibrinogen levels exceeding 5 g/l also increased the risk for postoperative PE. The higher the fibrinogen, the stronger the inflammatory reaction, and the faster the fibrin formation, the poorer its resolution [23].

Neither FVIII nor D-dimer were associated with postoperative PE, but elevated FVIII did indicate decreased survival. In addition, high FVIII may be a universal predictor of postoperative survival for patients with pathologic fractures, as it displayed limited variation between patients with different primary diseases. High FVIII was associated with overall mortality also in a larger population study investigating inflammatory diseases [24]. FVIII derives from endothelial activity, is carried by released von Willebrand factor, and enhances thrombin generation. D-dimer is the joint marker of both coagulation activity and fibrinolysis, and cancer has been associated with both enhanced coagulation and aberrant fibrinolytic system. Local tissue remodeling occurs at sites where fibrinolytic degradation takes part in tumor development and progression. Thus, the systemic fibronolytic balance is likely to be altered and fibrin resolution impaired in advancing cancer [25].

Literature on PE among patients with surgery for pathologic fractures is scarce, and the rates differ markedly between 2 and 8.5% [1, 8, 9, 12, 26]. When compared with the reported rates reported, our PE rate is slightly higher. However, we did not observe the adverse effects of PE on overall survival, which have been observed in other larger studies [8, 9]. Further, because on average PE occurred on post-operative day 36, the 1 month duration of thromboprophylaxis seems insufficient as these patients are in a procoagulant state, likely causing rebound activation of coagulation upon cessation of LMWH, while the preoperative role of fibrinogen and FVIII only gets stronger (OR 8.9) later on, for instance at 2 years.

Our observations may add to other scoring systems predicting survival when considering the method of surgery, since aggressive massive resection and large reconstruction could be avoided in those patients having limited benefit from the procedures. A combination of FVIII and fibrinogen may serve as a biomarker for postoperative survival, as together they could identify the patients who would be poor survivors. Although D-dimer is a well-known predictor of PE, D-dimer was elevated in our study in all patients with pathologic fractures and did not associate with survival or PE. The combination of fracture, exposure of blood to bone and cancerous tissue and surgical trauma may consume plasmin activity, and therefore fibrin may be inadequately degraded, as several metabolic states reflect on fibrinolysis [27].

Research on thromboembolism after orthopedic procedures of the upper extremities is limited. The rate of postoperative thromboembolism after humeral fractures, even without thromboprophylaxis, has been considered low [28], and the routine use of thromboprophylaxis following upper extremity trauma has not been recommended. A previous meta-analysis reported a rate of postoperative DVT of less than 1% and no postoperative PE in patients who underwent surgery for primary skeletal tumors of the upper extremity [1]. One study showed a similar rate of thromboembolic complications after intramedullary nailing of upper and lower extremity pathologic fractures of long bones [26]. Along systemic blood-bone interactions, such as coagulation activity, we show that the incidence of postoperative PE is similar irrespective of the site of skeletal metastases and fractures and suggest that thromboprophylaxis should be administered to all pathological fractures to avoid cancer-associated thrombosis.

Several limitations may have influenced the results of this cohort study. As the study design is retrospective and observational, selection bias may be an issue. The patients were, however, collected from two centers with centralized patient information systems, and the documentation of the patient information is standardized. Moreover, the treatment protocols, laboratory services, and indications for surgery of these two university clinics are similar. The patients had different primary diseases at different stages. Also, a minority of patients used anticoagulants preoperatively for other indications. The follow-up times for each patient varied. Fibrinogen, as an indicator of increased risk for PE, showed significant results in Kaplan-Meier analysis but not in Cox regression analysis, and these results need to be verified in larger patient groups. The potential biomarker use of fibrinogen to identify the need for a more aggressive and longer or a different thromboprophylactic protocol also demands further studies.

## Conclusions

The results of our study establish fibrinogen and FVIII, acute phase reactants promoting coagulation, as suitable preoperative biomarkers for postoperative survival and complications after the operative treatment of pathologic fractures. Overall, fibrinogen appears to be a more sensitive marker for postoperative survival and PE than FVIII, which was very high on average (exceeding 250 IU/dL). Fibrinogen, however, varies between patient groups with different primary diseases, and therefore the combination of fibrinogen and FVIII captured the outcome better across all cancer types. In addition, our results highlight the need for novel improved thromboprophylaxis strategies due to the high incidence of PE. Specifically, patients with metastatic fractures of the upper limb should be better protected from VTE complications. A randomized study setup for both pre- and postoperative compared with only postoperative anticoagulation or thromboprophylaxis would be of interest.

## Supplementary Information

**Additional file 1.**

## Data Availability

The datasets used and analyzed during the current study are available from the corresponding author on reasonable request.

## Abbreviations

APTT: Partial thromboplastin time
AT: Antithrombin
CCI: Modified Charlston comorbidity index
FVIII: Coagulation factor VIII
LMWH: Low-molecular-weight heparin
PE: Pulmonary embolism
PT: Prothrombin time
TT: Thrombin time
VTE: Venous thromboembolism
