# Supplementary material for: Preoperative coagulation biomarkers associate with survival and pulmonary embolism after surgical treatment of non-spinal skeletal metastases
Source: Thromb J. 2022 Nov 22;20:70. doi: 10.1186/s12959-022-00431-w (PMC9682700; doi:10.1186/s12959-022-00431-w)
Supplement: Supplementary file 1 — Additional file 1. [file 12959_2022_431_MOESM1_ESM.docx]

**Cox regression survival analysis of the combination of high fibrinogen and FVIII with confounding variables.**

|  | RR | 95 CI | p-value |
| --- | --- | --- | --- |
| **Fibrinogen >4 g/l and FVIII >326 IU/dl** | 3.33 | 1.68-6.62 | 0.001 |
| **Modified Charlston comorbidity index** | 0.65 | 0.05-9.27 | 0.752 |
| **Age over 65 years** | 1.76 | 0.80-3.85 | 0.158 |
| **Hypertension** | 1.76 | 0.93-3.31 | 0.081 |
| **Chemotherapy** | 1.25 | 0.65-2.39 | 0.498 |
| **Radiation treatment** | 1.48 | 0.50-4.38 | 0.479 |
| **Fracture** | 1.15 | 0.51-2.57 | 0.736 |
